# Bayesian Population-Based Prevalence Estimation of *KCNV2*-Associated Retinopathy: A Comparative Analysis of Disease-Associated Variant Frequencies in Russian and Global Populations

**DOI:** 10.3390/ijms27135911

**Published:** 2026-06-30

**Authors:** Anastasiia V. Rozhkova, Ekaterina A. Rutkovskaya, Vitaly V. Kadyshev, Anton A. Esibov, Almaqdad Alsalloum, Ekaterina S. Kuznetsova, Julia A. Krupinova, Olga N. Mityaeva, Kristina K. Shefer, Ernest V. Boiko, Olesya V. Sagaydak, Natalya A. Doroshchuk, Maria V. Makarova, Mary Woroncow, Viktor P. Bogdanov, Pavel Y. Volchkov

**Affiliations:** 1 Federal Research Center for Innovator and Emerging Biomedical and Pharmaceutical Technologies, Ministry of Science and Higher Education of the Russian Federation, 125315 Moscow, Russiavolchkov_py@academpharm.ru (P.Y.V.); 2 Moscow Center for Advanced Studies, Ministry of Science and Higher Education of the Russian Federation, 123592 Moscow, Russia; 3 Federal State Budgetary Scientific Institution “Research Centre for Medical Genetics named after Academician N.P. Bochkov”, Ministry of Science and Higher Education of the Russian Federation, Moskvorechye st., 1, Moscow 115522, Russia; 4 State Budgetary Institution of Healthcare Moscow Clinical Scientific and Practical Center named after A.S. Loginov Moscow City Health Department, Novogireevskaya st., bldg. 1, Moscow 111123, Russia; 5 Federal State Autonomous Institution “National Medical Research Center for Eye Microsurgery named after Academician S.N. Fyodorov” of the Ministry of Health of the Russian Federation, Saint Petersburg Branch, Yaroslav Gashek Str. 21, 192283 St. Petersburg, Russia; 6 Ophthalmology Department of North-Western State Medical University named after I.I. Mechnikov, Kirochnaya Str. 41, 191015 Saint-Petersburg, Russia; 7 National Medical Research Center of Cardiology named after Academician E.I. Chazov, 121552 Moscow, Russia; 8 Evogen LLC, 115191 Moscow, Russia; 9 Faculty of Medicine, Lomonosov Moscow State University, Leninskie Gory 1, 119991 Moscow, Russia

**Keywords:** *KCNV2*-associated retinopathy, orphan disease, bayesian estimation, population prevalence, population genomics, opthalmogenetics

## Abstract

Cone dystrophy with supernormal rod responses (CDSRR) is a rare autosomal recessive hereditary retinal dystrophy caused by biallelic *KCNV2* variants. Accurate prevalence data remain limited because current estimates rely on clinically ascertained cases. This study aimed to estimate its population prevalence by integrating curated variant evidence and large-scale population genomics resources. The key methodological feature of this study is a multi-tiered pathogenicity framework combined with estimation of a biologically plausible prevalence range: Known pathogenic variants define conservative estimates, whereas probably pathogenic variants and strong variants of uncertain significance define broader estimates. Allele frequencies were analyzed in 807,162 individuals from the gnomAD database and 144,127 Russian genomes from GDB and EvogenDB. A Bayesian framework was applied to calculate disease prevalence from aggregated carrier frequencies across predefined variant tiers. Conservative estimates based on known pathogenic variants, were 1 in 343,926 globally and 1 in 1,911,347 in Russia. Including broader tiers increased estimates to 1 in 14,620 and 1 in 32,764, respectively. Compared to previous clinically ascertained prevalence of 1 in 865,000, these estimates define a wider biologically plausible prevalence range and are consistent with possible clinical underascertainment. This tier-based approach accounts for uncertainty in variant classification when estimating rare disease prevalence.

## 1. Introduction

Cone dystrophy with supernormal rod responses (CDSRR; OMIM #610356) is a rare inherited retinal disorder caused by biallelic mutations in the *KCNV2* gene, which encodes the voltage-gated potassium channel modifier subfamily V member 2 [1,2]. CDSRR is clinically characterized by a constellation of progressive visual symptoms, typically with onset in childhood. These symptoms reflect predominant cone photoreceptor dysfunction with secondary rod involvement, leading to distinctive functional impairment [3]. Furthermore, pronounced photophobia is a cardinal symptom, stemming directly from cone dysfunction and manifesting as significant discomfort or avoidance of brightly lit environments [4]. CDSRR is predominantly inherited in an autosomal recessive manner, requiring two mutant alleles—one inherited from each asymptomatic carrier parent [5].

The most recent meta-analysis, conducted by de Guimaraes et al. [6], encompasses 22 studies that identified 95 unique pathogenic *KCNV2* variants in the reported cases. Missense substitutions constitute the largest class of mutations (~48%), followed by nonsense and frameshift mutations (collectively ~41%) [6]. A significant proportion of variants (~2/3) affect the gene’s amino-terminal region (N-terminus and NAB domains), highlighting its critical role [6]. Additionally, a recent genotype–phenotype study reported that patients with biallelic missense variants consistently showed superior visual acuity and less structural deterioration [7]. Beyond the retinal phenotype, isolated case reports have suggested potential associations between *KCNV2* variants and other conditions, including atrioventricular nodal reentry tachycardia [8], developmental disorders [9], and epilepsy [10].

To date, a single founder mutation (c.427G > T; p.Glu143Ter) has been well-documented, specifically in the Arab population [11]; however, the genetic architecture of the disease suggests that additional population-specific founder variants likely exist but remain unreported. Furthermore, a hypomorphic variant (c.854T > G, p.Met285Arg) has also been associated with the disease [12]. It is postulated to cause a mild or subclinical phenotype in the homozygous state. Consequently, this variant is likely pathogenic only in compound heterozygosity with a more deleterious allele [12].

Accurate prevalence estimates are crucial for healthcare resource planning and for designing adequately powered clinical trials. For rare diseases, such as *KCNV2*-associated retinopathy, current estimates typically rely on clinical ascertainment, which may be affected by underdiagnosis, misdiagnosis, and incomplete molecular confirmation, thereby maintaining significant uncertainty in burden-of-disease assessments. The emergence of large, open-access population genomics databases provides a powerful alternative approach, enabling the estimation of autosomal recessive disease prevalence from carrier frequencies at the population level [13,14]. In this study, we leverage these resources through a multi-tiered pathogenicity framework, combined with range-based prevalence estimation. Known pathogenic variants were used to generate conservative estimates, whereas probable pathogenic variants and strong variants of uncertain significance were incorporated to define broader estimates. This tier-based approach accounts for uncertainty in variant classification and provides a biologically plausible prevalence range, moving beyond single-point estimates based solely on clinical data.

## 2. Results

### 2.1. Tiered KCNV2 Variant Spectrum for Prevalence Estimation

**Figure 1:**
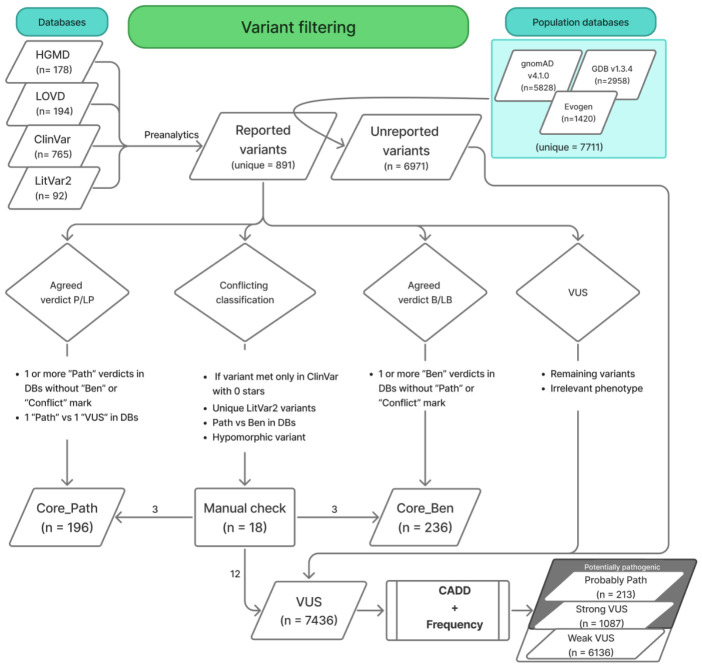
Overview of the tier-based KCNV2 variant framework used for prevalence estimation. Variants were compiled from curated clinical databases, the published literature, and population genomic resources, and assigned to predefined evidence groups: core pathogenic variants, core benign variants, and variants of uncertain significance (VUS). The VUS group was further stratified into probably pathogenic variants, strong VUS, and weak VUS according to the classification strategy described in Methods. Arrows indicate the direction of variant selection, filtering, and stratification. Colors and background shading are used to visually group related categories and indicate the sets to which summary counts apply. The label “n” denotes the number of unique genetic variants in the corresponding group or set. Three nested variant sets were used for prevalence estimation: core pathogenic variants only; core pathogenic plus probably pathogenic variants; and core pathogenic plus probably pathogenic variants plus strong VUS, jointly referred to as potentially pathogenic variants.

The overall tier-based framework used to generate variant sets for prevalence estimation is summarized in Figure 1. Briefly, known pathogenic and benign *KCNV2* variants were used to define reference groups, while remaining rare variants were stratified into predefined evidence tiers. This framework generated three nested prevalence scenarios, ranging from conservative estimates based on core pathogenic variants to broader estimates incorporating probable pathogenic variants and strong variants of uncertain significance.

According to the study design, data extraction from public databases yielded 196 core pathogenic variants and 1300 potentially pathogenic variants. The potentially pathogenic dataset consisted of 213 probably pathogenic variants and 1087 strong VUS. A complete list of pathogenic and potentially pathogenic variants, along with their frequencies, is provided in Supplementary File S1.

**Figure 2:**
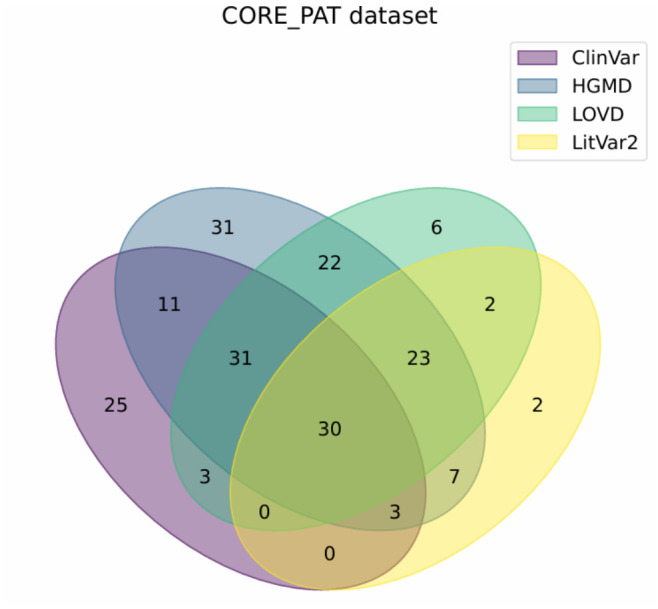
Overlap of core pathogenic *KCNV2* variants across curated sources. The Venn diagram shows the intersections between core pathogenic variant sets derived from HGMD, ClinVar, LOVD, and published literature. Each primary color corresponds to one of the four sources, while overlapping regions indicate variants shared between two or more sources. The incomplete overlap between sources indicates that no single resource captured the full curated pathogenic variant spectrum used in this study.

The core pathogenic variants were compiled from HGMD [15], ClinVar [16], LOVD [17], and published literature, with incomplete overlap between sources (Figure 2). This indicates that no single database captured the full curated spectrum of pathogenic variants for *KCNV2* and supports the use of multiple complementary sources when constructing variant sets for population-based prevalence estimation.

**Figure 3:**
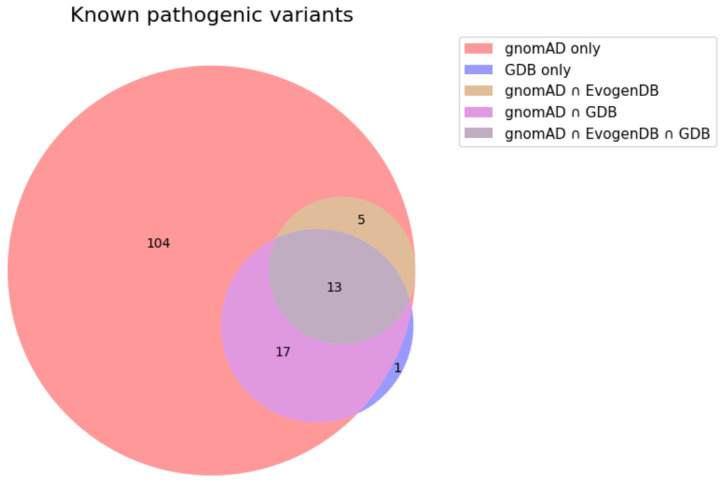
Representation of core pathogenic variants in population frequency databases. Among the 196 core pathogenic variants, 140 were observed in at least one population database. Most pathogenic variants detected in Russian genomic databases were also represented in gnomAD. One reported pathogenic variant, chr9-2718845-T-TG, was identified exclusively in the Russian population datasets, with an allele frequency of 0.000003.

Among the 196 core pathogenic variants, 140 were represented in at least one population frequency database and therefore contributed observed allele counts to the prevalence estimates (Figure 3). This finding indicates that a substantial proportion of reported pathogenic *KCNV2* variants are absent from available population datasets, highlighting the dependence of population-based prevalence estimation on database coverage.

**Figure 4:**
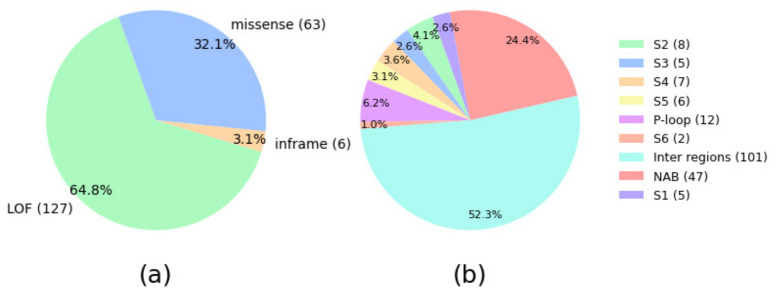
Mutational spectrum and protein-domain distribution of core pathogenic *KCNV2* variants. (**a**) Distribution of variants by effect type. LOF group contains nonsense, frameshift, canonical splice-site and start-loss variants. (**b**) Distribution of variants according to the affected protein domain. The pie chart represents 193 of the 196 identified variants. The remaining three variants are splice-site variants that could not be mapped to the protein sequence: c.1356 + 3_1356 + 6del, c.1357-1G > A, c.1357-1G > C. Percentages in panel (**b**) sum to 99.9% due to rounding.

Figure 4a shows the mutational spectrum in our dataset, which differs from the mutational spectra reported in previous studies. While previous reports indicate missense variants constitute 48% and loss-of-function (nonsense and frameshift) variants account for 41% of the total, our curated dataset showed a different distribution: loss-of-function variants account for 64.8% of known pathogenic variants and missense variants account for 32.1%. Mapping of variants onto protein domains is largely consistent with the previous study, supporting the functional importance of key structured regions [6]. However, the currently available expanded mutational spectrum, with variants from the past five years incorporated, indicates that the majority of pathogenic changes are located in interdomain regions, rather than within the core domains themselves (Figure 4b). Together, these findings indicate that the apparent pathogenic spectrum of *KCNV2* mutation is sensitive to ongoing variant discovery and database updating, which is relevant for variant curation and prevalence estimation.

**Figure 5:**
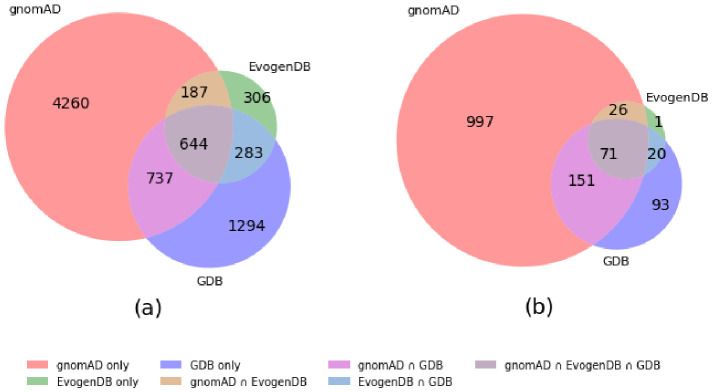
Overlap of *KCNV2* variants across population genomic databases. (**a**). A Venn diagram showing the overlap of all distinct *KCNV2* variants retrieved from gnomAD, GDB, and EvogenDB; (**b**). A Venn diagram showing the overlap of core pathogenic and potentially pathogenic variants represented in these population databases.

Population genomic databases contributed the largest pool of rare *KCNV2* variants for tier-based assessment. The number and overlap of distinct *KCNV2* variants retrieved from gnomAD [18], GDB [19], and EvogenDB [20] are shown in Figure 5a. After tier-based classification, the final potentially pathogenic set included 1300 variants, comprising 213 probably pathogenic variants and 1087 strong VUS (Supplementary File S1). Comparison of core pathogenic and potentially pathogenic variants across population datasets revealed incomplete overlap between global and Russian genomic resources (Figure 5b). These dataset-specific differences in variant representation support the relevance of population-specific variant spectra for prevalence estimation.

### 2.2. Tier-Based Estimates of CDSRR Prevalence

Observed allele frequencies for core pathogenic variants were available for 36 variants in the combined Russian population databases GDB and EvogenDB and for 139 variants in gnomAD v4.1.0. The remaining core pathogenic variants were not observed in these population datasets and therefore did not contribute to the observed allele counts in the prevalence estimates. Using the Bayesian model described in Materials and Methods, conservative prevalence estimates based on core pathogenic variants were 1 in 343,926 globally and 1 in 1,911,347 in Russia (Table 1). The prevalence of *KCNV2*-associated retinopathy was estimated using the Bayesian model described in the Section 4. The corresponding expected numbers of affected individuals were then calculated for the Russian population (146 million) and the worldwide population (8 billion).

To evaluate the effect of variant-classification uncertainty on prevalence estimation, we next estimated prevalence across the predefined nested variant tiers (Table 2). Inclusion of probably pathogenic variants increased the estimated prevalence compared with the conservative core pathogenic set. Further expansion of the variant set to include strong VUS generated the broadest estimates, reaching 1 in 14,620 globally and 1 in 32,764 in Russia. The most inclusive estimate also included the previously reported hypomorphic *KCNV2* variant, as specified in the variant inclusion criteria. Thus, the tier-based analysis produced a prevalence range from conservative estimates based on known pathogenic variants to broader estimates incorporating potentially pathogenic variants. This pattern shows that prevalence estimates for *KCNV2*-associated retinopathy are highly sensitive to the variant set used for calculation.

### 2.3. Bayesian Re-Assessment of Prevalence Using Patient Cohort Data

To refine the prevalence estimate, we incorporated variants from our patient cohort into the core pathogenic variant set and performed a re-analysis. Clinical and genetic information was available for 15 patients with molecularly confirmed CDSRR, who had been evaluated at the Moscow Research Center for Medical Genetics (Supplementary File S2).

Seventeen distinct *KCNV2* variants were identified in these patients, including five variants that had not previously been reported as pathogenic or likely pathogenic in the literature or public databases (Table 3). For these five variants, allele frequencies were higher in the Russian population databases than in gnomAD. Since the contribution of each allele to the total frequency varies with sample size, we also provide the absolute allele counts in parentheses to mitigate this scaling effect. These data suggest that variants from our patient cohort may contribute more substantially to Russian population-based estimates than to globally aggregated estimates.

The updated prevalence estimates are presented in Table 4. The incorporation of additional data led to an increase in point estimates of prevalence and a corresponding widening of the Bayesian credible intervals: the interval width increased by 0.6% for the gnomAD-based core pathogenic estimate and by 11.7% for the estimate based on aggregated Russian population frequency databases.

Consequently, the inclusion of patient-cohort-derived variants had a greater impact on Russian prevalence estimates than on gnomAD-based estimates. This pattern is consistent with the presence of a population-specific spectrum of *KCNV2* pathogenic variants. However, the magnitude of this effect should be interpreted in the context of the substantially lower initial Russian prevalence estimate, population database size, allele representation, and variant-classification uncertainty.

### 2.4. Population-Specific Distribution of KCNV2 Variants in the Russian and gnomAD Databases

**Figure 6:**
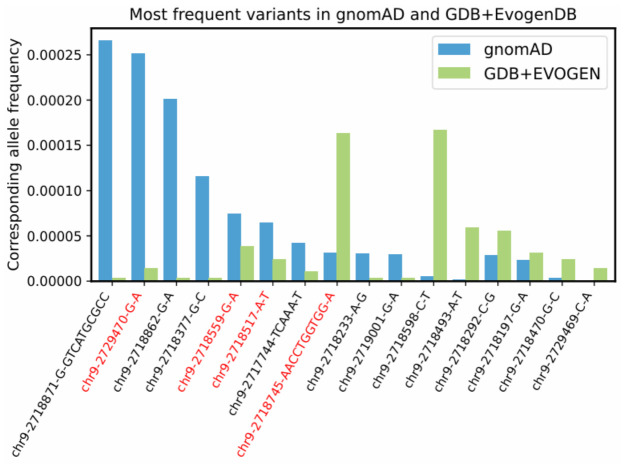
Comparison of the ten most frequent *KCNV2* variants in gnomAD and the combined Russian population database. Variants present in the top-10 lists of both datasets are highlighted in red. Limited overlap between the two lists indicates dataset-specific differences in the allele-frequency spectrum of *KCNV2* variants.

Figure 6 shows a comparison of the ten most common variants in the gnomAD database and the combined Russian database. Notably, only four variants are shared between the two top-10 lists, indicating differences in the allele-frequency spectrum between globally aggregated and Russian population datasets. Within our patient cohort, the two most common variants were chr9:2718598C > T (present in 6 alleles, 4 individuals, including 2 homozygotes) and chr9:2718493A > T (present in 4 alleles, 3 individuals, including 1 homozygote). Both of these variants are also ranked among the top-10 frequent variants in the combined Russian database. In contrast, none of the additional patient-cohort-derived variants listed in Table 3 were present among the most frequent variants in either database.

## 3. Discussion

In this systematic analysis, we used allele frequencies from gnomAD to estimate the worldwide prevalence of *KCNV2*-associated retinopathy and Russian databases to determine prevalence specifically in Russia. Our estimates range from 1 in 343,926 to 1 in 14,620 globally and from 1 in 1,911,347 to 1 in 32,764 in Russia. Even the most conservative gnomAD-based estimate was approximately 2.5-fold higher than the previously reported clinically derived prevalence of 1 in 865,000 [21]. Although direct comparison between model-based and clinically ascertained estimates should be interpreted with caution, this difference provides population-genetic evidence consistent with systematic clinical underascertainment of *KCNV2*-associated retinopathy.

That previous estimate was derived by extrapolating data from a clinical cohort at the University of Iowa Department of Ophthalmology Retina Clinic (January 2010–June 2016), where CDSRR accounted for 2 cases per 1000 patients [21]. A key limitation of this approach is the non-random, referral clinic-based nature of the cohort, despite its broad geographic representation across 40 of the 50 U.S. states.

*KCNV2*-associated retinopathy is difficult to diagnose due to its clinical heterogeneity. A definitive diagnosis requires demonstration of biallelic pathogenic *KCNV2* variants, together with pathognomonic electroretinographic findings, constituting the current diagnostic gold standard. Estimates based on clinically identified cases may therefore substantially underestimate disease prevalence, as they do not account for individuals with mild, atypical, or misdiagnosed manifestations who remain without molecular confirmation. For these reasons, the use of additional prevalence estimation methods not based on clinical diagnosis may be useful for changing diagnostic strategies.

Population-based genetic data are increasingly being used to estimate the prevalence of rare autosomal recessive disorders from the aggregate frequencies of pathogenic variants. Schrodi et al. developed a general framework for estimating the prevalence of monogenic autosomal recessive diseases using population genetic data [13]. A more recent study applied a similar approach to GNE myopathy and demonstrated its value for reassessing the prevalence of an underdiagnosed rare disorder [14]. These studies illustrate the growing role of large population sequencing databases as a complementary source of epidemiological evidence, particularly when estimates based on clinically ascertained cohorts are limited by underdiagnosis, referral bias, or restricted access to molecular testing.

A key feature of the present study is the use of a multi-tiered pathogenicity framework combined with range-based prevalence estimation. The conservative estimates were calculated using core pathogenic variants only, whereas broader estimates incorporated probably pathogenic variants and strong variants of uncertain significance. This design makes variant-classification uncertainty explicit and provides a biologically plausible prevalence range. The marked increase in prevalence estimates after expansion of the variant set demonstrates that rare disease prevalence modeling is highly sensitive to pathogenicity assumptions. However, the broader estimates should not be interpreted as definitive prevalence values, because inclusion of uncertain variants may lead to overestimation if some candidates are later reclassified as benign.

The construction of the core pathogenic variant set also demonstrated that no single curated source captured the full pathogenic variant spectrum of *KCNV2*. Core pathogenic variants were distributed across HGMD, ClinVar, LOVD, and the published literature, with incomplete overlap between sources. This supports the use of multiple complementary resources when constructing variant sets for population-based prevalence estimation. Reliance on a single database could lead to exclusion of relevant disease-associated variants and, consequently, to underestimation of genetic prevalence.

Another important limitation of population-based prevalence estimation is the extent of database coverage. Among the 196 described core pathogenic variants, gnomAD contained population-frequency data for 139 variants, whereas a total of 36 variants were found in the aggregated Russian databases GDB and EvogenDB. The remaining variants were not observed in these population datasets and therefore did not contribute observed allele counts to prevalence estimation. Their absence may reflect extreme rarity, limited sample size, underrepresentation of relevant populations, technical differences in sequencing or variant calling, or incomplete representation of private and population-specific alleles. Thus, population-based prevalence estimates depend not only on the curated pathogenic variant spectrum but also on the coverage and representativeness of the population frequency databases used.

This updated curated pathogenic variant set differs from previously reported mutational spectra. In our dataset, loss-of-function variants accounted for 64.8% of known pathogenic variants, whereas missense variants accounted for 32.1%. This shift may reflect ongoing variant discovery, differences in curation criteria, reporting bias, or increased recognition of truncating and splice-disrupting variants. These findings suggest that the mutational spectrum of *KCNV2*-associated retinopathy is still incompletely characterized and may change as additional patients are diagnosed, molecular testing becomes more widely used, and new disease-associated variants are reported. Mapping variants onto protein domains further showed that pathogenic changes were not confined to core-structured domains. This supports comprehensive gene-wide variant assessment, although the functional relevance of variants outside canonical domains requires additional evidence.

Estimates from the combined Russian population databases were substantially lower than global estimates when only core pathogenic variants were included. In addition to differences in database size and allele representation, this may reflect a population-specific spectrum of *KCNV2* pathogenic variants. The comparison of the ten most frequent *KCNV2* variants in gnomAD and the combined Russian database further supports this interpretation: only four variants were shared between the two top-10 lists, indicating differences in the allele-frequency spectrum between globally aggregated and Russian population datasets. However, such differences should not be interpreted solely as true differences in disease prevalence between populations. They may also reflect sample size, ancestry composition, database structure, variant-calling differences, and unequal representation of rare alleles.

In our data, population-specific frequency information was particularly relevant for interpreting the Russian estimates. Two of the most common variants in the patient cohort were also among the most frequent *KCNV2* variants in the combined Russian database, supporting the relevance of local population data for variant prioritization and prevalence estimation. Conversely, rare patient-cohort-derived variants that were absent or extremely rare in major population databases could not be fully captured by population-frequency-based modeling alone, potentially leading to underestimation of genetic prevalence under the model assumptions. These observations underscore the importance of complementing in silico prevalence estimates with up-to-date clinical genetic data.

Interestingly, a well-described founder mutation for the Arab population, c.427G > T (p.Glu143Ter), illustrates the limitations of globally aggregated population databases. In gnomAD v4.1.0, this variant is present with a frequency of 0.000006 [11]. A total of 8 alleles were detected in the European non-Finnish population (allele count: 1,179,598) and 1 allele in the Middle Eastern population (allele count: 6060). This observation highlights that even comprehensive databases are not equally representative of all populations. Underrepresentation of specific populations, including those from Russia and the Middle East, may limit the detection and frequency estimation of population-specific disease-associated variants.

Together, these observations support a broader methodological point: globally aggregated reference databases may not fully capture local variant architectures, particularly for rare recessive disorders in populations that are unevenly represented in international genomic resources. For *KCNV2*-associated retinopathy, this was illustrated by the limited overlap between the most frequent variants in gnomAD and Russian databases and by the greater impact of patient-cohort-derived variants on Russian prevalence estimates. These findings highlight the value of population-specific genomic resources for variant prioritization, diagnostic interpretation, and rare disease prevalence modeling.

From a clinical perspective, these findings may inform diagnostic strategy, variant prioritization, and approximate patient population sizing for *KCNV2*-associated retinopathy. A broader and population-aware *KCNV2* variant spectrum may support more systematic interpretation of rare variants detected in patients with atypical or incomplete CDSRR phenotypes and may help refine diagnostic workflows for inherited retinal dystrophies. Broader access to molecular genetic testing, together with awareness of characteristic electroretinographic findings, may improve case ascertainment and facilitate the identification of patients who could be eligible for natural history studies or future gene-based therapeutic trials. However, these applications require cautious interpretation because the estimates remain dependent on variant classification, database coverage, and model assumptions.

Several additional limitations should be considered. First, structural variants, including CNVs and other complex rearrangements, were not included in the calculations. This may lead to incomplete estimates, particularly for the conservative lower-bound scenario. Second, we assumed that pathogenicity classifications in genetic databases were accurate, which may not always be the case. Third, when analyzing variants of uncertain significance, we assumed that these variants cause *KCNV2*-associated retinopathy, rather than only other phenotypes with which they may have been reported. Fourth, only one hypomorphic variant was included in the most comprehensive estimate. Given the clinical variability of CDSRR, additional variants with hypomorphic effects may exist but remain insufficiently characterized. Finally, we relied on CADD scores to define groups of potentially pathogenic variants. Although the cutoffs were chosen based on sensitivity and specificity for *KCNV2*, this approach can produce both false-positive and false-negative results.

The Hardy–Weinberg equilibrium assumption represents an additional limitation of the present analysis. Non-random mating, including consanguinity and endogamy, may increase the frequency of individuals carrying biallelic disease-associated variants compared with that expected under random mating; this effect was not incorporated into the present prevalence model. Differences in allele frequencies among ethnic, geographic, or relatively isolated subpopulations, together with founder effects, may introduce an additional source of bias, as pooled allele frequencies can obscure locally increased frequencies of population-specific variants. Therefore, the results reported in this study should be interpreted as average pooled estimates and may differ substantially from the actual prevalence in particular ethnic or regional populations.

This limitation is particularly relevant to the multi-ethnic population of the Russian Federation. However, the available Russian databases do not contain sufficiently detailed ancestry, regional, pedigree, or kinship information to derive reliable subgroup-specific allele frequencies or to estimate the level of inbreeding represented in the analyzed sample. Consanguinity and endogamy are not uniformly distributed across the Russian population and are more characteristic of particular ethnic or regional groups, which represent only a part of the overall population, whose contribution to the available reference datasets cannot be reliably quantified. Accordingly, applying an inbreeding coefficient to the pooled Russian dataset would substantially complicate interpretation without providing greater accuracy, as such an adjustment would not be supported by population-specific empirical data.

The calculation also assumes that rare disease-associated variants are effectively independent, as only aggregate AC and AN data were available; individual-level co-occurrence and phase could not be assessed. If two variants occur in cis on the same chromosome copy, as part of the same haplotype, summing their allele counts may count that chromosome twice and thereby overestimate the aggregate frequency of disease-associated alleles. Although such co-occurrence is expected to be uncommon for most rare variants, complex alleles and linked variants inherited together from a common ancestor may represent important exceptions. Finally, the reported credible intervals reflect uncertainty associated with the finite number of observed alleles under the specified Bayesian model, but do not account for population structure, non-random mating, founder effects, linkage disequilibrium, penetrance, or uncertainty in variant classification.

## 4. Materials and Methods

### 4.1. Data Sources: Identification and Curation of KCNV2 Variants

Sources of genetic variants in the *KCNV2* gene were grouped into two broad categories:

1. Sources of variants with a known clinical effect;
2. Sources of variants with an unknown clinical effect.

Extraction of variants from databases was performed in July 2025.

Databases of clinical variants were mined to obtain the most comprehensive list of reported disease-associated *KCNV2* variants. Three curated variant databases were used: HGMD Professional (QIAGEN GmbH, Hilden, Germany) [15], ClinVar (National Center for Biotechnology Information, National Library of Medicine, National Institutes of Health, Bethesda, MD, USA) [16], and Leiden Open Variation Database v3.0 (LOVD; Leiden University Medical Center, Leiden, the Netherlands) [17]. In total, 197 variants were retrieved from HGMD, 765 from ClinVar, and 194 from LOVD. Structural variants were excluded, and the analysis was restricted to SNVs and short indels. Entries were then filtered by phenotype, retaining variants relevant to *KCNV2*-associated retinopathy and related retinal phenotypes. Variants associated exclusively with non-target phenotypes were not included in the core pathogenic set and were assigned to the variant-of-uncertain-significance (VUS) group for subsequent assessment.

Submission-level classifications were harmonized into the following categories: pathogenic, benign, VUS, and, for ClinVar, conflicting pathogenicity. Variants with conflicting or ambiguous evidence were reviewed manually. The full consensus classification rules, phenotype filters, and database-specific extraction procedures are provided in Supplementary File S3.

Literature-derived variants were retrieved using LitVar2 (National Center for Biotechnology Information, National Library of Medicine, National Institutes of Health, Bethesda, MD, USA) [22], a text-mining tool that extracts SNVs and short indels from PubMed and PMC records. Because automated text-mining tools may misextract or incorrectly annotate variants, all variants retrieved only through LitVar2 and not supported by curated databases were subjected to manual verification. After expert review of titles, abstracts, and, when necessary, full texts, 245 *KCNV2*-associated articles were narrowed to 111 relevant publications. LitVar2 yielded 92 variants, 9 of which were not found in HGMD, ClinVar, or LOVD. Since LitVar2 does not assign pathogenicity classifications, these variants were annotated as literature-reported variants and evaluated manually.

Variants with conflicting or ambiguous evidence underwent expert manual review, including variants with conflicting pathogenicity verdicts, variants with zero-star ClinVar submissions, and variants detected only using LitVar2. After manual review, 3 variants were assigned to the core pathogenic group, 3 to the core benign group, and 12 to the VUS group. Detailed manual review decisions are provided in Supplementary File S3.

All variants identified through database and literature searches were normalized to a unified format, validated against the GRCh38 genome assembly, deduplicated, and mapped to the NM_133497.4 transcript, which was used as the canonical *KCNV2* reference sequence. Variant coordinates and transcript annotations were checked using Ensembl REST API queries (EMBL-EBI, Hinxton, UK); cDNA-to-protein HGVS conversion was performed using pyhgvs v.0.11.1 (open-source Python package); and the canonical transcript was confirmed using MANE Select GRCh38 v1.4 (NCBI, Bethesda, MD, USA, and EMBL-EBI, Hinxton, United Kingdom; accessed July 2025).

Population frequency databases were used as the primary source of additional rare *KCNV2* variants of uncertain significance. Population frequency data were obtained from Genome Aggregation Database v4.1.0 (gnomAD; Broad Institute of MIT and Harvard, Cambridge, MA, USA) [18], the Russian Genetic Diversity Database (GDB v1.3.4; Federal Medical-Biological Agency of Russia, Moscow, Russia) [19], and EvogenDB, a non-public institutional population genomic database maintained by Evogen LLC (Moscow, Russia) [20]. gnomAD was used as a globally aggregated population genomic resource comprising 730,947 exomes and 76,215 whole genomes. GDB and EvogenDB were used as Russian population genomic resources and included whole-genome sequencing data from 120,762 and 23,365 Russian participants, respectively. The Russian databases represent the multi-ethnic composition of the Russian Federation; however, detailed ancestry- or region-specific data are not publicly available because of privacy and ethical restrictions.

Because GDB and EvogenDB are independent datasets, they were combined for the Russian population analysis by summing allele counts and allele numbers for each variant. The combined allele frequency for each variant was calculated as the sum of allele counts divided by the sum of allele numbers. Analyses were performed separately for gnomAD and for the combined Russian population database.

To conclude this stage, we extracted all variants along with their allele frequencies from population databases. These data were assigned to the corresponding variants in our list of reported variants. Any variant from the population datasets that did not match an entry in the reported list was assigned to the VUS group for subsequent analysis. As a result, all variants were classified into one of three distinct groups: Core pathogenic (196 variants), Core benign (236 variants), or VUS (7436 variants).

### 4.2. Variant Filtering and Pathogenicity Assessment

During the literature review, the variant *KCNV2*:854T > G (p.Met285Arg, rs148050307), previously described as hypomorphic [12], was identified. Because this variant is not expected to cause *KCNV2*-associated retinopathy in the homozygous state alone, it was not included in the core pathogenic group. To avoid overestimating the conservative lower-bound prevalence, it was retained outside the core pathogenic set. However, because its disease association has been reported in compound-heterozygous combinations with more severe variants, it was included in the most comprehensive prevalence scenario. The expected contribution of hypomorphic homozygotes was excluded from the prevalence estimate, whereas its potential contribution in compound-heterozygous combinations was retained.

All variants obtained from population frequency databases and not included in the curated pathogenic or benign lists underwent additional filtering using Combined Annotation Dependent Depletion v1.7 for GRCh38 scores (CADD v1.7; Berlin Institute of Health at Charité, Berlin, Germany, and University of Washington, Seattle, WA, USA; accessed July 2025) [23] and allele-frequency thresholds. Variants of uncertain significance were considered potentially pathogenic if they had an allele frequency below 0.005, consistent with ACMG/AMP recommendations for autosomal recessive diseases [24], and a CADD score of at least 15 [25].

**Figure 7:**
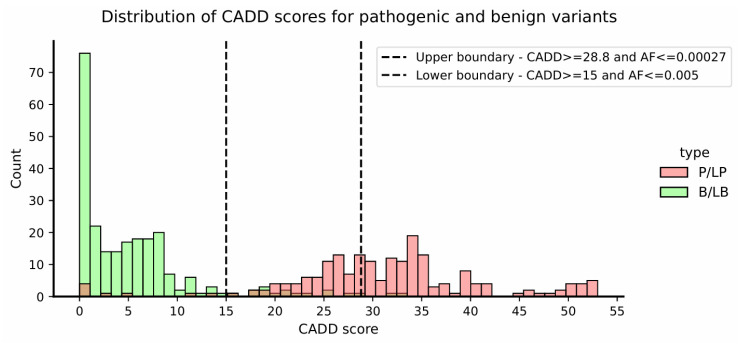
Distribution of CADD scores among curated core pathogenic and core benign *KCNV2* variants used as reference sets for threshold selection. The overlap between the two distributions illustrates the limited ability of CADD alone to fully separate pathogenic and benign variants. The selected cutoffs of 15 and 28.8 were used to define strong VUS and probably pathogenic variants, respectively, in combination with allele-frequency thresholds.

Potentially pathogenic variants were then stratified into probably pathogenic and strong VUS using two CADD cutoffs combined with allele-frequency ceilings (Figure 7). Probably pathogenic variants required CADD scores of 28.8 or higher and allele frequency no greater than 0.00027, corresponding to the allele frequency of the most common known pathogenic *KCNV2* variant in gnomAD. This threshold prioritized high specificity (99.2%) and a low false discovery rate (1.6%). Strong VUS were defined as variants with CADD scores of 15 or higher and allele frequency below 0.005, unless they met criteria for the probably pathogenic group. This lower threshold prioritized broader inclusion of potentially disease-associated variants. The derivation of CADD thresholds, distribution of CADD scores in reference pathogenic and benign variant sets, confusion matrices, sensitivity, specificity, false discovery rate, and threshold-stability analyses are provided in Supplementary File S3.

As a result of applying this strategy, three groups of variants were obtained in order of decreasing confidence in pathogenicity:

(1) Core pathogenic variants in the *KCNV2* gene;
(2) Probably pathogenic variants with CADD ≥ 28.8 and AF ≤ 0.00027;
(3) Strong VUS with CADD ≥ 15 and AF ≤ 0.005.

These variant lists (Supplementary File S1) were applied in different combinations to calculate disease prevalence rates.

### 4.3. Prevalence Estimation

Prevalence estimates of *KCNV2*-associated retinopathy for both the global population and Russia were derived using a Bayesian framework based on the approach described by Schrodi et al. [13]. This approach is particularly suited to rare disease epidemiology, as it formally integrates observed genetic data with prior evidence to obtain posterior estimates under conditions of uncertainty.

In this Bayesian model, the final prevalence estimate is taken as the expectation of the posterior probability distribution of *q*^2^, where *q* represents the aggregate frequency of disease-associated *KCNV2* alleles. This generates a data-driven probability estimate for disease frequency. We applied this procedure separately to three distinct variant sets to establish a prevalence spectrum:

1. Core pathogenic variants only (defining a conservative lower bound);
2. Core pathogenic + probably pathogenic variants;
3. Core pathogenic + probably pathogenic + strong VUS (defining a plausible upper bound).

For each variant set, the allele counts of all variants included in the corresponding set were summed to obtain the observed number of disease-associated alleles in the reference population sample (*x*). The total number of evaluated alleles was represented by *2n*. For the Russian databases, *2n* was calculated as twice the number of individuals whose data were included in the respective database. For gnomAD, the variant-specific AN reported at each genomic position was used, as it reflects the number of alleles that were successfully evaluated at that position and may therefore be lower than twice the total number of individuals represented in the database. The aggregate allele frequency *q* estimated from the observed number of disease-associated alleles was used to calculate the expected frequency of individuals carrying two disease-associated alleles. Under the Hardy–Weinberg equilibrium assumption, the expected genetic prevalence of individuals carrying two disease-associated alleles was represented by *q*^2^:

(1) $${(p}^{2}+2pq+\;q^{2}=\;1)$$

This value, based on the observed count of causative alleles in the reference population sample (x), was used to model the posterior density of the allele frequency. After obtaining the most probable prevalence based on known frequency data, we calculated a 95% credible interval:

(2) $$E(q^{2}|x;2n)=\frac{(2n-1)!}{2(2n-x-1)!(x-1)!}\int_{0}^{1}q^{\frac{x}{2}}{\left(1-\sqrt{q}\right)}^{2n-x-1}dq$$

For the known hypomorphic variant, which is not expected to cause *KCNV2*-associated retinopathy in the homozygous state alone, the expected contribution of hypomorphic homozygotes was excluded from the prevalence estimate, whereas its potential contribution in compound-heterozygous combinations with more severe variants was retained.

The model assumed Hardy–Weinberg equilibrium, random mating, and effective independence of rare disease-associated alleles. Original aggregate AC and AN values were used without adjustment for population structure, relatedness, founder effects, or inbreeding. Individual-level phase information was unavailable; therefore, the possible co-occurrence of multiple variants in cis could not be directly assessed. The reported credible intervals reflect uncertainty associated with the finite number of observed alleles under the specified Bayesian model, but do not incorporate uncertainty arising from population stratification, non-random mating, founder effects, linkage disequilibrium, penetrance, or variant classification.

All Bayesian prevalence calculations were performed using custom Python scripts in Python v3.10.8 (Python Software Foundation, Wilmington, DE, USA) using the Jupyter extension for Visual Studio Code v2024.11.0 (Microsoft Corporation, Redmond, WA, USA). The Jupyter Notebook containing all analytical code, software versions, and package dependencies is available in Supplementary File S4.

### 4.4. Patients

This study presents a cohort of 15 patients from 14 families with clinically and molecularly confirmed *KCNV2*-associated retinopathy, who were clinically evaluated by our research group. Patients 12 and 13 are siblings. Patients 14 and 15 have been reported in our previous publication [26]. The genetic and clinical characteristics of these cases are summarized in Supplementary File S2. The studies involving humans were approved by the Ethics Committee of the Research Center for Medical Genetics. The studies were conducted in accordance with local legislation and institutional requirements. The patient’s parents provided written informed consent to participate in this study.

All study participants underwent targeted next-generation sequencing (NGS) using a custom panel for inherited retinal diseases at the Research Center for Medical Genetics (Moscow, Russia). Genomic DNA was isolated from peripheral blood samples of the patients and, where available, their family members, using the ExtractDNA Blood Kit (Evrogen JSC, Moscow, Russia) according to the manufacturer’s protocol. Variants were mapped and annotated using the reference transcript NM_133497.4 as the canonical sequence.

Variants identified in the patient cohort were used to re-assess disease prevalence and to characterize the *KCNV2* variant spectrum in clinically confirmed CDSRR cases. Patient-cohort-derived variants considered causative in clinically and molecularly confirmed cases and not previously included in the core pathogenic reference set were added to the core pathogenic variant list. The expanded core pathogenic variant set was then used to recalculate prevalence estimates using the same Bayesian framework. The previously selected CADD thresholds of 15 and 28.8 were retained; recalculated sensitivity, specificity, and false discovery rate after expansion of the positive reference set are provided in Supplementary File S3.

## 5. Conclusions

In this study, we aimed to characterize the range of genetic prevalence estimates for *KCNV2*-associated retinopathy, from the most conservative estimate based on core pathogenic variants to more inclusive estimates incorporating potentially pathogenic variants selected according to formal criteria. The most conservative estimates were 1 affected individual in 343,926 in the pooled gnomAD dataset and 1 in 1,911,347 in the combined Russian databases, whereas the most inclusive estimates were 1 in 14,620 and 1 in 32,764, respectively. The conservative gnomAD-based estimate was approximately 2.5-fold higher than the previously reported clinically derived prevalence of 1 in 865,000. Although direct comparison between model-based and clinically ascertained estimates should be interpreted with caution, this finding is consistent with the possibility that *KCNV2*-associated retinopathy remains underdiagnosed.

Our results also highlight the importance of considering the population-specific spectrum of variants in the gene of interest when estimating disease prevalence. The inclusion of variants identified in the Russian patient cohort had a more pronounced effect on the Russian estimate than on the pooled gnomAD estimate, suggesting that global reference databases may not fully capture rare variants relevant to particular populations. However, the reported values represent pooled genetic prevalence estimates and may differ from the actual prevalence in specific ethnic or regional populations because of differences in population structure, variant representation, sample composition, and the assumptions incorporated into the model.

These findings may support diagnostic planning, population-aware variant prioritization, and future study design for *KCNV2*-associated retinopathy. Broader access to molecular genetic testing, together with increased awareness of characteristic electroretinographic findings among ophthalmologists, may contribute to improved case ascertainment. More complete identification of affected individuals would facilitate further refinement of prevalence estimates, improve understanding of the natural history of the disease, and support the development of future clinical studies and gene-based therapeutic approaches.

These findings should nevertheless be interpreted in light of the remaining limitations of the study, particularly uncertainty in variant classification, the exclusion of structural variants, and the assumptions of Hardy–Weinberg equilibrium and effective independence of rare disease-associated alleles.

## Figures and Tables

**Table 1:** Calculated prevalence based on core pathogenic variants.

Characteristics	Core Pathogenic Variants	Credible Interval
	**gnomAD v4.1.0**	
Affected 1/over	1/343,926	1/319,523–1/370,985
Affected globally	23,261 affected people	21,564–25,037 affected people
	**GDB v1.3.4 + EvogenDB**	
Affected 1/over	1/1,911,347	1/1,476,909–1/2,544,652
Affected in Russia	76 affected people	57–99 affected people

**Table 2:** Prevalence estimates across expanded *KCNV2* variant tiers.

Characteristics	Core Pathogenic Variants + Probably Pathogenic Variants	Core Pathogenic Variants + Probably Pathogenic Variants + Strong VUS
	**gnomAD v4.1.0**	
Affected 1/over	1/177,864	1/14,620
Credible interval	1/167,084–1/189,632	1/14,131–1/15,133
Affected globally	44,978 affected people	547,192 affected people
Credible interval	42,187–47,880 affected people	528,656–566,116 affected people
	**GDB v1.3.4 + EvogenDB**	
Affected 1/over	1/688,082	1/32,764
Credible interval	1/562,248–1/856,499	1/29,761–1/36,204
Affected in Russia	212 affected people	4456 affected people
Credible interval	170–260 affected people	4033–4906 affected people

**Table 3:** Novel variants identified in the Russian CDSRR patient cohort.

No	Variant	gnomAD: Allele Frequency (Allele Count)	GDB + EvogenDB: Allele Frequency (Allele Count)	CADD_Score	Use in Calculations
1	chr9-2718584-T-C	-	0.00001 (3)	24.7	Strong VUS
2	chr9-2718695-T-C	0.000001 (2)	-	27.1	Strong VUS
3	chr9-2718704-C-A	0.000001 (2)	0.000021 (6)	15.66	Strong VUS
4	chr9-2718514-GCCAAGGCCATCGGGGTGGCCT-G	0.0000006 (1)	0.000003 (1)	17.1	Strong VUS
5	chr9-2718501-C-A	0.000004 (6)	0.000021 (6)	13.22	NO

**Table 4:** Re-evaluated disease prevalence.

Characteristics	Core Pathogenic Variants	Core Pathogenic Variants + Probably Pathogenic Variants	Core Pathogenic Variants + Probably Pathogenic Variants + Strong VUS
**gnomAD v4.1.0**			
Affected 1/over	1/341,193	1/176,846	1/14,607
Credible interval	1/317,031–1/367,981	1/166,143–1/188,529	1/14,119–1/15,119
Affected globally	23,447 affected people	45,237 affected people	547,688 affected people
Credible interval	21,740–25,234 affected people	42,434–48,151 affected people	529,140–566,624 affected people
**GDB v1.3.4 + EvogenDB**			
Affected 1/over	1/1,648,613	1/628,841	1/32,518
Credible interval	1/1 285,297–1/2,170,990	1/516,070–1/778,794	1/29,543–1/35,926
Affected in Russia	89 affected people	232 affected people	4490 affected people
Credible interval	67–114 affected people	187–283 affected people	4064–4942 affected people

## Data Availability

Publicly available databases: gnomAD v4.1.0 (https://gnomad.broadinstitute.org/, accessed on 31 July 2025), ClinVar (https://www.ncbi.nlm.nih.gov/clinvar/, accessed on 31 July 2025), LOVD (https://www.lovd.nl/, accessed on 31 July 2025), LitVar2 (https://www.ncbi.nlm.nih.gov/research/litvar2/, accessed on 31 July 2025). Restricted-access databases: HGMD (https://digitalinsights.qiagen.com/, accessed on 31 July 2025), GDB FMBA v1.3.4 (https://gdbpop.nir.cspfmba.ru/, accessed on 31 July 2025), EvogenDB (https://evogenlab.ru/en/, accessed on 31 July 2025). Patient`s data: The raw clinical and genetic data from patients are not publicly available due to privacy protections and restrictions imposed by the informed consent agreements. Derived datasets provided in this study: We provide three preprocessed datasets containing *KCNV2* variants: (1) “Core_Pathogenic”—variants classified as pathogenic/likely pathogenic; (2) “Core_Benign”—variants classified as benign/likely benign; (3). “Uncertain”—variants of uncertain significance (VUS) or variants without a definitive pathogenicity assertion in the source databases. All variants in these datasets are de-identified: information regarding the specific source database for each variant has been removed. This approach respects the licensing requirements of the original databases, including HGMD, whose data cannot be redistributed without explicit permission. Additionally, we provide a complete list of all variants available in the gnomAD database, along with their respective frequencies. These data files are made available for analysis via a Jupyter notebook. Furthermore, we include a results file containing core pathogenic variants, probably pathogenic variants, and strong VUS, along with their frequencies in gnomAD. Code availability: A Jupyter notebook containing the complete code for reproducing all analyses is provided in the Supplementary Materials. The notebook can be used together with the provided datasets (Core_Pathogenic, Core_Benign, Uncertain) to verify all calculation results. Reproducibility: All data processing and statistical analysis steps are described in detail in the Methods Section. When replicating this study, it should be noted that the databases used (both open-access and restricted) may be updated with new information over time; therefore, future replications may yield slightly different values compared to those presented in this work.

## Supplementary Materials

The following supporting information can be downloaded at: https://www.mdpi.com/article/10.3390/ijms27135911/s1.

## Abbreviations

The following abbreviations are used in this manuscript:

ACMG/AMP	American College of Medical Genetics and Genomics/Association for Molecular Pathology
API	Application Programming Interface
CADD	Combined Annotation-Dependent Depletion
cDNA	Complementary Deoxyribonucleic Acid
CDSRR	Cone Dystrophy with Supernormal Rod Responses
ClinVar	Public Archive of Relationships Between Human Variations and Phenotypes
CNVs	Copy Number Variations
GDB	The Russian Genetic Diversity Database
gnomAD	Genome Aggregation Database
GRCh38	Genome Reference Consortium Human Build 38
HGMD	Human Gene Mutation Database
LOVD	Leiden Open Variation Database (Leiden Open-source Variation Database)
NAB	N-terminal A box
NGS	Next-Generation Sequencing
SNVs	Single-Nucleotide Variants
VUS	Variant of Uncertain Significance
