# Supplementary material for: Bayesian Population-Based Prevalence Estimation of KCNV2-Associated Retinopathy: A Comparative Analysis of Disease-Associated Variant Frequencies in Russian and Global Populations
Source: Int J Mol Sci. 2026 Jun 30;27(13):5911. doi: 10.3390/ijms27135911 (PMC13361675; doi:10.3390/ijms27135911)
Supplement: Supplementary file 1 [file ijms-27-05911-s001.zip › ijms-4369183-supplementary/Additional files/Supplementary File 3. Supplementary Methods.pdf]

**This document contains detailed information on the materials and methods section.**

Table of contents


1. Phenotype-based filtering of clinically reported KCNV2 variants. 2
2. Consensus classification of ClinVar submissions. 3
3. Cross-database integration algorithm for final variant classification. 4
4. Detailed Descriptions and Evidence Review for Conflicting Variants. 4
5. Population frequency database handling. 15
6. CADD-based filtering of uncertain variants. 15
7. CADD threshold-performance and stability analysis. 17
8. Re-evaluation with patient-cohort-derived variants. 19
9. Change in Bayesian credible interval width. 20

## 1. Phenotype-based filtering of clinically reported *KCNV2* variants.

Clinical-variant databases were mined to obtain the most comprehensive list of reported disease-associated KCNV2 variants. Phenotype-based filtering, retaining only entries relevant to our disease of interest, was applied separately to HGMD and ClinVar. Entries annotated exclusively with an unrelated phenotype were reassigned to the VUS. Lists of included and excluded phenotype terms:

**HGMD included phenotype terms:**

- "Cone dystrophy with supernormal rod responses";
- "Cone-rod dystrophy";
- "Cone rod dystrophy";
- "Cone|cone-rod dystrophy";
- "Retinopathy";
- "Retinal disease";
- "Cone dystrophy";
- "Rod-cone dystrophy";
- "Retinal dystrophy";
- "Retinitis pigmentosa";
- "Macular dystrophy, occult";
- "Impaired visual acuity";
- "Retinal cone dystrophy, type 38";
- "High myopia, early-onset (eoHM)";
- "Retinal degeneration".

**HGMD excluded phenotype terms:**

- "Developmental disorder";
- "Atrioventricular nodal reentry tachycardia";
- "Epilepsy";
- "Leber congenital amaurosis";
- "Parkinson disease, early-onset" ;

- “Cerebellar ataxia, mental retardation & dysequilibrium syndrome”.

**ClinVar  included (relevant) phenotype terms:**

- “Cone-rod dystrophy”;
- “Retinal dystrophy”;
- “Cone dystrophy with supernormal rod response”;
- “KCNV2-related disorder”;
- “Cone-rod dystrophy 6”;
- “Cone dystrophy”;
- “cone dystrophy with supernormal rod electroretinogram”;
- “inborn genetic disease”;
- “not provided”;
- “not specified”.

## 2. Consensus classification of ClinVar submissions.

Each germline variant in ClinVar may carry several submitted classifications, assessed against ACMG/AMP guidelines, which can disagree. To assign one consensus label per variant, the following rules were applied:

1. Definitely pathogenic or likely pathogenic verdicts in a database were unified to the “Pathogenic” label;

2. Ratio of pathogenic to VUS submits more than 2:1 were defined as “Pathogenic”;

3. Definitely benign or likely benign verdict in the database were unified to the “Benign” label;

4. Ratio of benign to VUS submits more than 2 : 1 were defined as “Benign”;

5. “VUS”  was defined when the VUS verdict or ratio of non-indifferent submits to VUS was 1:2;

6. “Conflicting pathogenicity” was defined as a submission having 0 stars.

Variants were thereby sorted into Pathogenic, Benign, VUS, and Conflicting groups; the Conflicting group underwent expert manual review.

## 3. Cross-database integration algorithm for final variant classification.

A final consensus classification was assigned to each unique variant by integrating the calls from HGMD, ClinVar, and LOVD into one of three groups. The following algorithm was implemented:

● Core pathogenic group (contains known pathogenic variants):

1. The variant was annotated as "Pathogenic" in at least one database, and no database contained a "Benign" or "Conflicting pathogenicity" call;
2. It was defined "Pathogenic" in HGMD, while in one of the other databases it is annotated as VUS. This prioritization of HGMD reflects its status as a manually curated repository of disease-associated variants.

● Core benign group: contains known benign variants. Symmetric rules were applied for this group, requiring at least one "Benign" call and the absence of any "Pathogenic" assertions.

● Variant of Uncertain Significance (VUS) group:

**1.** Variants associated exclusively with irrelevant disease (HGMD, ClinVar) were automatically assigned to this group;

**2.** Variants that were annotated as VUS exclusively in LOVD, ClinVar, or both, with no other pathogenicity calls;

**3.** Variants had a "Pathogenic" call solely in HGMD but VUS calls from both ClinVar and LOVD (1:2 evidence ratio). This approach downgraded variants with limited supportive pathogenic evidence to VUS for subsequent filtering.

## 4. Detailed Descriptions and Evidence Review for Conflicting Variants.

Variants with conflicting or ambiguous evidence underwent expert manual review. This group included variants with conflicting pathogenicity verdicts across curated databases, variants with zero-star ClinVar submissions, and variants detected only through LitVar2 without support from other curated clinical variant databases.

After deduplication, 18 unique variants underwent manual review. Final decisions were based on database assertions, reported phenotype, literature evidence, variant type, zygosity when available, and consistency with *KCNV2*-associated retinopathy. As a result, 3 variants were assigned to the core pathogenic group, 3 variants were assigned to the core benign group, and 12 variants were retained in the VUS group.

**Variants that were included in the Core_Pathogenic:**

| № | Variant (HG38) | PMID |
|---|---|---|
| 1 | chr9-2718625-CCA-C<br>NM_133497.4:c.887_888del<br>p.Pro296Argfs*75 | 30927187 |

**Verdict:** remain in the Core_Pathogenic set.

Reason: Null variant in a gene where LOF is a known mechanism of disease.

Description: The authors of the article describe a patient with CRD, who was found to be compound heterozygous for two mutations: c.655G>T and c.887_888del. Only the c.887_888del variant was found in her unaffected mother.

| № | Variant (HG38) | PMID |
|---|---|---|
| 2 | chr9-2718605-CG-C<br>NM_133497.4:c.870del<br>p.Gln291ArgfsTer31 | 26755771 |

**Verdict:** remain in the Core_Pathogenic set.

Reason: Null variant in a gene where LOF is a known mechanism of disease.

Description: The article PMID:26755771 refers to PMID:17896311. The authors of the article describe a patient with CRD, who was found to be compound heterozygous for two mutations: p.Lys260X (c.778A>T, This variant also found in another unrelated patient) and p.Ser289fs (c.867delC).

This article was published in 2007, since then the nomenclature rules have changed. Now this variant is identified as *KCNV2*(NM_133497.4):c.870del, p.Gln291ArgfsTer31.

| № | Variant (HG38) | PMID |
|---|---|---|
| 3 | chr9-2718859 C>T<br>NM_133497.4:c.1120C>T<br>p.Gln374Ter | PMID is absent |

**Verdict:** remain in the Core_Pathogenic set.

Reason: Null variant in a gene where LOF is a known mechanism of disease.

Description: This variant is classified as Pathogenic based on one submission to ClinVar (Variation ID: 3249226; submitted Jan 01, 2022; 0-star review status) by the Institute of Human Genetics, University of Regensburg. The variant is a null variant (nonsense, frameshift, canonical ±1 or 2 splice sites, initiation codon, single or multi-exon deletion) in a gene where loss of function (LOF) is a known mechanism of disease. Therefore, despite a single submitter entry, the variant remains in the Core set based on the established LOF evidence.

**Variants that were included in the Core_Benign:**

| № | Variant (HG38) | PMID |
|---|---|---|
| 1 | chr9-2717819-G-A<br>NM_133497.4:c.80G>A<br>p.Arg27His<br>rs145731729 | 23885164,<br>31180159,<br>34426522 |

**Verdict**: remain in the Core_Benign set.

Reason: Unable to accurately assess pathogenicity for complex allele

Description: The *KCNV2*:c.80G>A variant was reported in the study by Fujinami et al. (PMID: 23885164, 2013) as part of a complex allele with *KCNV2*:c.617 G>C, p.Arg206Pro, which is described as pathogenic in a patient with CRD [PMID: 36284460].

However, the available data do not provide convincing evidence supporting its pathogenicity. The functional impact of this variant has not been experimentally validated, and segregation data are lacking. Also, modern tools for working with population databases (gnomAD, GDB) do not allow for a reliable assessment of the prevalence of a complex allele.

The following articles (PMID:31180159,2019; PMID:34426522, 2021) refer to the study by Fujinami et al. (PMID: 23885164, 2013) and cannot be used as evidence of pathogenicity.

The variant is classified into the Core_Benign set for the following reasons. Its allele frequency is 0.13% in the gnomAD database, and it has been observed in the homozygous state in four individuals. Furthermore, this variant has 5 submissions with benign/likely benign verdicts in the ClinVar database (Variation ID: 143162), with no conflicting pathogenic submissions, although three entries are of uncertain significance. Supporting this, multiple in silico predictors assess the variant as benign.

| № | Variant (HG38) | PMID |
|---|---|---|
| 2 | chr9-2718386-T-C<br>NM_133497.4:c.647T>C<br>p.Ile216Thr<br>rs558295858 | 22264887,<br>34426522 |

**Verdict:** remain in the Core_Benign set

Reason: Variant found in patient does not have strong enough evidences to be considered as P/LP.

Description: This variant is not presented in the available text of the study (PMID:22264887). A later publication (PMID:34426522, 2021) cites the Thiadens et al. study (PMID:22264887, 2012) in a general context but does not provide any specific evidence — functional, segregation, or otherwise — to support pathogenicity of this variant. Therefore, this reference cannot be used as evidence for classification.

The variant is classified into the Core_Benign set for the following reasons. Its allele frequency is 0.10% in the gnomAD database, and it has been observed in the homozygous

state in six individuals. Furthermore, this variant has 2 submissions with benign/likely benign verdicts in the ClinVar database (Variation ID: 1167900), with one entry of uncertain significance and no conflicting pathogenic submissions.

| № | Variant (HG38) | PMID |
|---|---|---|
| 3 | chr9-2729602-G-T<br>NM_133497.4:c.1513G>T<br>p.Ala505Ser<br>rs192224431 | 27579568 |

**Verdict**: remain in the Core_Benign set.

Reason: Variant reported in the article is found in patient with different phenotype, not retinopathy one.

Description: The authors of the article (PMID:27579568) specify the absence of pathognomonic changes characteristic of CDSRR: “While the ERG in our patient did not show hyper response with increasing light exposure thresholds, suspicion remains regarding the contribution of the *KCNV2* mutation to the patient’s macular findings”. Thus, there is no sufficient basis for including this variant in the Core_Pathogenic set.

The variant is classified into the Core_benign set for the following reasons. Its allele frequency is 0.02% in the gnomAD database, with no homozygous individuals observed. Futhermore, this variant has a single submission with a likely benign verdict (1-star review status) in the ClinVar database (Variation ID: 1571757), dated October 15,2025.

**Variants that were defined in the Uncertain set:**

| № | Variant (HG38) | PMID |
|---|---|---|
| 1 | chr9-2718593 T>G<br>NM_133497.4:c.854T>G<br>rs148050307 | 38454848 |

**Verdict**: define in the Uncertain set.

Reason: Variant is classified into the Uncertain set to avoid overestimation of its allelic frequency and clinical impact.

Description: The variant is classified into the Uncertain set for the purpose of this analysis. Although population data (0.2% allele frequency, five homozygotes in gnomAD) and ClinVar submissions (Variation ID: 776404, two benign calls, including one submission dated after the publication of functional study - PMID:38454848) would suggest a benign interpretation, a functional study (PMID:38454848) demonstrates that this variant is a disease-causing when combined with a loss-of-function allele. Given the conflicting evidence, the hypomorphic nature of the variant, and the lack of data on clinical outcomes when it is combined with missense or in-frame variants, we have elected to classify it as a variant of uncertain significance and track its trajectory throughout the study.

| № | Variant (HG38) | PMID |
|---|---|---|
| 2 | chr9-2717738-C-T<br>NM_133497.4:c.-2C>T<br>rs75316505 | 22025579,<br>32483926 |

**Verdict**: define in the Uncertain set.

Reason (PMID: 22025579): Variant found in patient does not have strong enough evidences to be considered as P/LP.

Description: The *KCNV2*(NM_133497.4):c.-2C>T variant is located in the 5'UTR, potentially affecting normal splicing of the transcript. However, its current evidence for pathogenicity is limited. The variant is found in the gnomAD 4.1.0 with a frequency of 0.04%. This variant has been reported in the literature only once, in a patient with cone-rod dystrophy (CRD), in heterozygous state, without the identification of a second pathogenic allele in *KCNV2* (PMID: 22025579). Given that CRD caused by *KCNV2* follows an autosomal recessive inheritance pattern, the presence of the variant in a single heterozygous state does not fulfill the criteria for disease causation.

No functional studies have been published to confirm an effect on mRNA splicing or protein function, no segregation analysis in affected families has been reported.

Reason (PMID:32483926): Variant found in patient does not have strong enough evidences to be considered as P/LP.

Description: The *KCNV2*(NM_133497.4):c.-2C>T variant was observed in a heterozygous state. According to ClinVar (Variation ID:: 100603), this variant is currently classified as Likely Benign with a review status of 0 stars (last reviewed Feb 2023, 1 submission). However, based on the design of our study, which prioritizes inclusivity for variants with any level of uncertain or conflicting evidence, this variant was assigned to the VUS (variant of uncertain significance) group.

| № | Variant (HG38) | PMID |
|---|---|---|
| 3 | chr9-2718461-C-A<br>NM_133497.4:c.722C>A<br>p.Pro241Gln<br>rs765249083 | 26755771 |

**Verdict**: remove from the Core set.

Reason: Artifact of LitVar2 machine learning text mining.

Description: The article (PMID:26755771) refers to an earlier study (PMID:17896311) in which the authors describe a patient with cone-rod dystrophy carrying two adjacent heterozygous single-nucleotide changes: c.721C>T and c.722C>A. Analysis of the proband's unaffected mother revealed the same two changes in cis configuration, indicating that they reside on the same allele and together result in a nonsense substitution — p.(Pro241Ter). No second pathogenic allele was identified in this patient. These two variants should be considered together as a single multinucleotide variant (MNV): chr9:2718460-CC-TA. This MNV is already included in the Core pathogenic variant set.

| № | Variant (HG38) | PMID |
|---|---|---|

| | | |
|---|---|---|
| 4 | chr9-2729561-T-A<br>NM_133497.4:c.1472T>A<br>p.Ile491Asn<br>rs765182129 | 33546218 |

**Verdict**: remove from the Core set.

Reason: Artifact of LitVar2 machine learning text mining.

Description: Variant *KCNV2*(NM_133497.4):c.1472A>T is absent in this article.

| № | Variant (HG38) | PMID |
|---|---|---|
| 5 | chr9-2718323-C-T<br>NM_133497.4:c.584C>T<br>p.Thr195Met<br>rs750543416 | 33546218 |

**Verdict:** remove from the Core set.

Reason: Artifact of LitVar2 machine learning text mining.

Description: Variant *KCNV2*(NM_133497.4):c.584C>T is absent in this article.

| № | Variant (HG38) | PMID |
|---|---|---|
| 6 | chr9-2717757-G-C<br>NM_133497.4:c.18G>C<br>p.Glu6Asp<br>rs1436024276 | 26755771 |

**Verdict:** remove from the Core set.

Reason: Artifact of LitVar2 machine learning text mining.

Description: Variant *KCNV2*(NM_133497.4):c.18G>C is absent in this article.

| № | Variant (HG38) | PMID |
|---|---|---|
| 7 | chr9-2718464-A-G<br>NM_133497.4:c.725A>G<br>p.Gln242Arg<br>rs1306668241 | 26755771 |

**Verdict:** remove from the Core set.

Reason: Variant found in patient does not have strong enough evidences to be considered as P/LP.

Description: The article (PMID:26755771) cites an earlier study (PMID:21882291) in which the authors considered the potential pathogenicity of rare variants based solely on their low frequency, without providing functional or segregation data to support these claims. In the present case, the variant's classification as potentially pathogenic relies primarily on its rarity, which is insufficient evidence for assignment to the pathogenic or likely pathogenic category under current standards.

| № | Variant (HG38) | PMID |
|---|---|---|
| 8 | chr9-2718161-A-G<br>NM_133497.4:c.422A>G<br>p.Asp141Gly<br>rs1004802068 | 33546218 |

**Verdict:** remove from the Core set.

Reason: PMID:35457050 - Artifact of LitVar2 machine learning text mining.

Description: Variant *KCNV2*(NM_133497.4):c.422A>G is absent in this article.

| № | Variant (HG38) | PMID |
|---|---|---|

| | | |
|---|---|---|
| 9 | chr9-2718200-A-C<br>NM_133497.4:c.461A>C<br>p.Asp154Ala | 32967234 |

**Verdict:** remove from the Core set.

Reason: Artifact of LitVar2 machine learning text mining.

Description: Variant *KCNV2*(NM_133497.4):p.D154A is absent in this article.

| № | Variant (HG38) | PMID |
|---|---|---|
| 10 | chr9-2718494-A-G<br>NM_133497.4:c.755A>G<br>p.Lys252Arg | 39792073 |

**Verdict**: remove from the Core set.

Reason: Variant found in patient does not have strong enough evidences to be considered as P/LP.

Description: In the article, the variant is reported using the cDNA notation c.755G>A and the protein notation p.(Ala259Thr). However, according to the *KCNV2* reference transcript (NM_133497.4), the nucleotide substitution corresponding to the p.Ala259Thr amino acid change is c.775G>A. The designation c.755G>A does not align with the reference sequence and likely represents a reporting error. Based on the correct sequence alignment, the variant was reassigned to its proper nomenclature — *KCNV2*(NM_133497.4):c.775G>A (p.Ala259Thr) — which is already present in the Core pathogenic variant set and was therefore retained there.

The variant corresponding to the originally reported cDNA position — *KCNV2*(NM_133497.4):c.755A>G — represents a different substitution and was evaluated separately. In the absence of functional evidence, segregation data, or literature support, this variant was assigned to the VUS (variant of uncertain significance) group in accordance with the design of our study.

| № | Variant (HG38) | PMID |
|---|---|---|
| 11 | chr9-2718047-T-C<br>NM_133497.4:c.308T>C<br>p.Val103Ala<br>rs1586686718 | PMID is absent |

**Verdict**: remove from the Core set.

Reason: Variant found in patient does not have strong enough evidences to be considered as P/LP.

Description: The *KCNV2*(NM_133497.4):c.308T>C (p.Val103Ala) variant is a missense substitution affecting a conserved amino acid residue. It is currently classified as Likely pathogenic in ClinVar (ID: 3249970, 1 submission, 0 stars, reviewed Jan 2019). However, no functional studies or segregation data have been published, and the variant is not reported in the peer-reviewed literature. Due to the limited evidence, this variant does not meet the criteria for pathogenic or likely pathogenic classification under current ACMG/AMP guidelines and was therefore assigned to the VUS group in our study.

| № | Variant (HG38) | PMID |
|---|---|---|
| 12 | chr9-2718737-G-T<br>NM_133497.4:c.998G>T<br>p.Ser333Ile | PMID is absent |

**Verdict**: remove from the Core set.

Reason: Variant found in patient does not have strong enough evidences to be considered as P/LP.

Description: The *KCNV2*(NM_133497.4):c.998G>T (p.Ser333Ile) variant is a missense substitution affecting an amino acid residue within the functional domain of the protein. No functional studies, segregation data, or reports in the peer-reviewed literature have been published for this specific variant. It is currently classified as Likely pathogenic in ClinVar (ID: 3249717, 1 submission, 0 stars, reviewed Jan 2014), based on a single

submitter's assessment without detailed supporting evidence. In the absence of a second pathogenic allele in the patient, familial segregation data, or functional validation, this variant does not meet the criteria for pathogenic or likely pathogenic classification under current ACMG/AMP guidelines. Therefore, consistent with the design of our study, it was assigned to the VUS group.

## 5. Population frequency database handling.

Population frequency data were extracted from gnomAD v4.1.0, GDB v1.3.4, and EvogenDB. For the combined Russian population analysis, GDB and EvogenDB were treated as independent datasets. For each variant, allele counts and allele numbers were summed across the two Russian databases. The combined allele frequency was calculated as:

$$AF\_combined = (AC\_GDB + AC\_EvogenDB) / (AN\_GDB + AN\_EvogenDB).$$

For gnomAD, variant-specific AN values were used in prevalence calculations because the number of successfully evaluated alleles may differ between genomic positions.

## 6. CADD-based filtering of uncertain variants.

Combined Annotation Dependent Depletion (CADD) scores were used to stratify undescribed KCNV2 variants of uncertain significance and to define groups of potentially pathogenic variants. This section provides additional detail on the filtering algorithm summarized in the main text.

First, we defined the initial criteria under which a variant of uncertain significance could be considered potentially pathogenic: (1) allele frequency (AF) below 0.005, in accordance with ACMG/AMP recommendations for autosomal recessive diseases [24]; and (2) scaled CADD score of at least 15, following the recommendations of the developers of the predictive algorithm [25].

Scaled CADD scores were then compared between the curated core pathogenic and core benign KCNV2 variant sets, which were used as reference groups. Core pathogenic variants were treated as positive observations, whereas core benign variants were treated as negative observations. The distribution of CADD scores in these two reference groups is shown in Figure 7 of the main manuscript. For each evaluated CADD threshold, variants with scores equal to or above the threshold were classified as predicted pathogenic, and variants

with scores below the threshold were classified as predicted benign. Sensitivity was defined as the proportion of core pathogenic variants correctly identified as predicted pathogenic, specificity as the proportion of core benign variants correctly classified as predicted benign, and false discovery rate (FDR) as the proportion of false-positive classifications among all variants predicted as pathogenic.

A CADD threshold of 28.8 was selected for the probably pathogenic category because it provided high specificity of 99.2% and a low FDR of 1.6%, although the corresponding sensitivity was 64.3%. This stringent threshold therefore prioritized the exclusion of benign variants and reduced the number of false-positive classifications, at the cost of missing a proportion of pathogenic variants. The lower threshold of 15, retained for the strong VUS category, provided sensitivity of 95.9%, specificity of 92.0%, and an FDR of 9.2%. This lower threshold enabled broader inclusion of potentially disease-associated variants, while accepting a higher proportion of false-positive classifications. The confusion matrices for the selected thresholds of 15 and 28.8 are shown in Figure S1.

In addition to CADD-based criteria, variant categorization incorporated allele-frequency thresholds. The allele frequency of the most common known pathogenic KCNV2 variant in gnomAD, 0.00027, was used as the maximum frequency permitted for variants classified as probably pathogenic. Accordingly, variants with CADD scores of 28.8 or higher and AF no greater than 0.00027 were categorized as probably pathogenic. Variants with CADD scores of 15 or higher and AF below 0.005 were categorized as strong VUS, unless they met criteria for the probably pathogenic group. Together, the CADD and allele-frequency thresholds defined the boundaries used to stratify variants of uncertain significance into probably pathogenic variants and strong VUS.

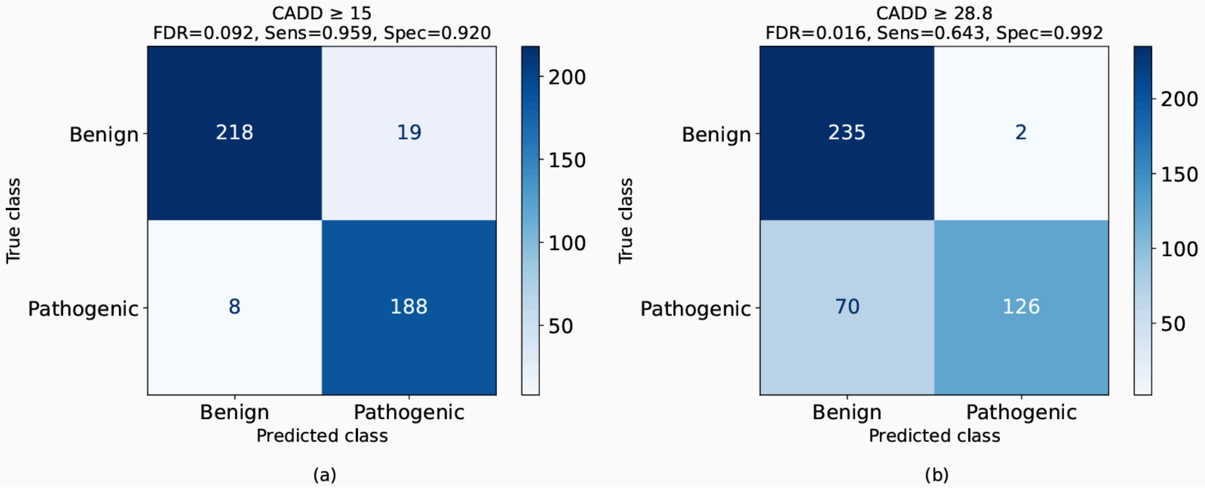

**Figure S1**. Confusion matrices for the CADD-based classification of KCNV2 variants. (a) Confusion matrix for the known pathogenic and benign reference variant sets using a CADD threshold of 15. (b) Confusion matrix for the same reference sets using a CADD threshold of 28.8. The false discovery rate (FDR), sensitivity (Sens), and specificity (Spec) are indicated for both thresholds.

## 7. CADD threshold-performance and stability analysis.

To evaluate the performance and stability of CADD-based variant stratification, the curated core pathogenic and core benign KCNV2 variant sets were used as reference groups. Core pathogenic variants were treated as positive observations, whereas core benign variants were treated as negative observations. For each evaluated CADD threshold, variants with scores equal to or above the threshold were classified as predicted pathogenic, and variants with scores below the threshold were classified as predicted benign. Sensitivity, specificity, and false discovery rate (FDR) were calculated for each threshold.

To assess the stability of the resulting variant stratification under modest changes in the selected CADD thresholds, the analysis was repeated using thresholds 2 CADD points below and 2 CADD points above the selected values. For the minimum threshold used to define strong VUS, CADD scores of 13, 15, and 17 were evaluated. For the threshold used to define probably pathogenic variants, CADD scores of 26.8, 28.8, and 30.8 were evaluated.

The resulting sensitivity, specificity, FDR, and variant counts are summarized in Table S1. For analyses of the upper CADD threshold, the reported variant count refers only to variants classified as probably pathogenic. For analyses of the lower CADD threshold, it represents the total number of variants included in the most inclusive variant set, comprising both probably pathogenic variants and strong VUS. Accordingly, changing the upper threshold alters the distribution of variants between these two categories, whereas changing the lower threshold alters the overall number of variants included in the expanded prevalence estimate.

Variants classified as probably pathogenic were required to have an allele frequency no greater than 0.00027, corresponding to the frequency of the most common known pathogenic *KCNV2* variant in gnomAD. Variants retained in the strong VUS category were required to have an allele frequency below 0.005.

**Table S1.** Sensitivity analysis of alternative CADD thresholds and their effect on KCNV2 variant stratification.

| CADD threshold | Sensitivity | Specificity | FDR | Number of analyzed variants | Prevalence gnomAD v4.1.0 | Prevalence GDB v1.3.4+EvogenDB |
|---|---|---|---|---|---|---|
| Lower CADD threshold | | | | | | |
| 13 | 0.964 | 0.907 | 0.104 | 1234 | 1/11 455 (1/11 099 - 1/11,827) | 1/14 135 (1/13 074 - 1/15 320) |
| 15 | 0.959 | 0.92 | 0.092 | 1073 | 1/14 620 (1/14 131 - 1/15 133) | 1/32 764 (1/29 761 - 1/36 204) |
| 17 | 0.954 | 0.928 | 0.083 | 917 | 1/22 972 (1/22 125 - 1/23 865) | 1/49,052 (1/44 111 - 1/54 795) |
| Upper CADD threshold | | | | | | |
| 26.8 | 0.709 | 0.983 | 0.028 | 358 | 1/97 579 (1/92 461 - 1/103 098) | 1/376,945 (1/316 533 - 1/454 562) |
| 28.8 | 0.643 | 0.992 | 0.016 | 209 | 1/177 864 (1/167 084 - 1/189 632) | 1/688,082 (1/562 248 - 1/856 499) |
| 30.8 | 0.531 | 0.992 | 0.019 | 133 | 1/251 543 (1/234 978 - 1/269 769) | 1/829 474 (1/671 525 - 1/1 043 857) |

The corresponding sensitivity and specificity profiles across the evaluated CADD thresholds, together with the receiver operating characteristic curve, are presented in Figure S3. The selected thresholds of 15 and 28.8 are indicated by dashed vertical lines. The area under the receiver operating characteristic curve was 0.965, indicating high overall discriminatory performance of CADD scores in the reference variant sets.

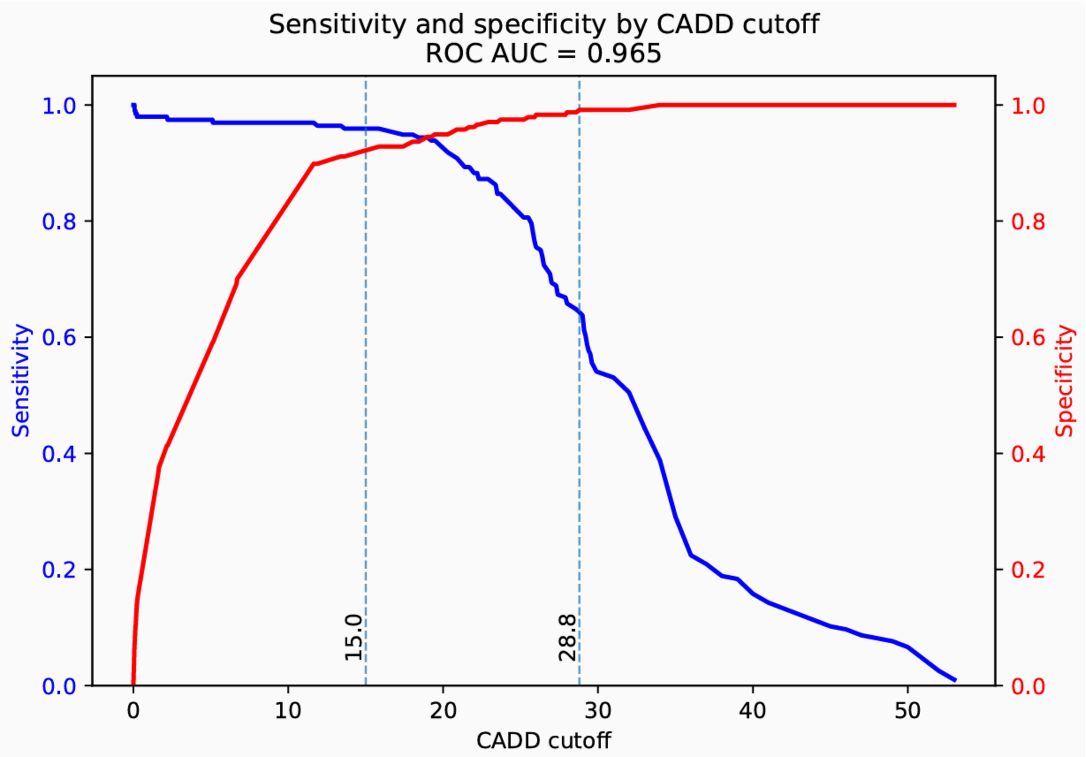


**Figure S3.** Sensitivity and specificity of CADD-based discrimination between core pathogenic and core benign KCNV2 variants across different CADD thresholds. Sensitivity and specificity are plotted as functions of the CADD cutoff. The selected thresholds of 15 and 28.8 are indicated by dashed vertical lines. The receiver operating characteristic curve is shown together with the corresponding area under the curve.

## 8. Re-evaluation with patient-cohort-derived variants.

Patient-cohort-derived KCNV2 variants considered causative in clinically and molecularly confirmed cases and not previously included in the core pathogenic reference set were added to the core pathogenic variant set. This expanded reference set was used to re-evaluate the performance of the previously selected CADD thresholds and to recalculate prevalence estimates.

The CADD thresholds of 15 and 28.8 were retained to ensure comparability with the primary analysis. However, because the composition of the positive reference set had changed, sensitivity, specificity, and false discovery rate were recalculated. At a CADD threshold of 28.8, sensitivity was 62.7%, specificity was 99.2%, and FDR was 1.6%. At a threshold of 15, sensitivity was 95.5%, specificity was 92.0%, and FDR was 9.0%. The expanded core pathogenic variant set was subsequently used to recalculate the prevalence estimates.

This re-evaluation was performed only to assess the effect of incorporating patient-cohort-derived variants and did not change the predefined CADD thresholds used for variant stratification.

## 9. Change in Bayesian credible interval width.

To quantify how uncertainty changed after incorporation of patient-cohort-derived variants, we calculated the percentage change in the width of the 95% Bayesian credible interval relative to the initial estimate. Credible interval width was calculated as the difference between the upper and lower bounds of the interval on the prevalence scale. The percentage change was calculated as:

$$Change = \frac{Credible\_interval\_width\_posterior - Credible\_interval\_width\_prior}{Credible\_interval\_width\_prior} * 100\%.$$

Positive values indicate widening of the credible interval after incorporation of patient-cohort-derived variants, whereas negative values indicate narrowing of the interval.
